# Remarks and Observations on Saccharine Diabetes, with the Treatment of Two Cases

**Published:** 1869-02

**Authors:** J. E. Manlove


### REMARKS AND OBSERVATIONS ON SACCHARINE DIABETES, WITH THE TREATMENT OF TWO CASES.

Read before the Nashville Medical Society, BY J. E. MANLOVE, M.D.

[Published by request.]

MR. PRESIDENT:—At our April meeting, the Society, at the suggestion of the author of this paper, selected for the consideration and discussion of the present meeting, the subject Diabetes; and, in obedience to the inexorable parliamentary law, I was appointed to open the discussion.

Prefatory to approaching this intricate and difficult subject, presenting as it does a vast field for physiological and pathological research and inquiry, allow me to say, that but two motives prompted me, in suggesting to the consideration of the Society:—1st. The highly satisfactory and encouraging results of the treatment of two cases, which have recently fallen under my observation; and, 2dly. The hope that some new light may be elicited and diffused, to the honor of our profession, and the mitigation and relief of human suffering. The treatment of the two cases referred to, though not entirely new, has certainly not been adopted or sufficiently insisted upon by the profession, in this country. Whether this has grown out of a misapprehension of its pathology, the infrequency of its occurrence, or the known intractable character of the disease, I know not; but certain I am, that the treatment hitherto has, to a great extent, been empirical, and productive of very little, if any, good and lasting results.

Before detailing the cases, with their treatment, the Society will indulge me in some preliminary remarks collaterally connected with this subject, and which are considered necessary to a full understanding and elucidation of it; together with the expression of my own poor opinion, and inferences deduced from the pathology of the disease.

The term Diabetes is derived from a Greek verb, which means to pass off, or through. Hence, by some, it has been called a urinary Diarrhœa; Diarrhœa urinosa, dipsas, diuresis, hydrops ad matulane, profluria urinæ, etc.; each name intending to imply an excessive discharge of crude urine, exceeding the quantity of fluid drank by the patient. Bœrhaave, in his Institutes, says: It is a frequent, copious discharge of lacteous urine, in conjunction with an extraordinary tenuity of the fluids.

Dr. Cullen places this genus of disease in the class neurosis, and order spasmi: which he defines a chronic flow of urine, in immoderate quantities, and of a preternatural quality. He notices two species:—1st. Diabetes Melletus, when the urine has the color, odor, and taste of honey. 2d. Diabetes Insipidus, when the urine is limpid only. Home defines it to be an extraordinary increase of urine, and that of a sweetish taste, attended with perpetual thirst and a dry skin.

The bare mention of the names of the above-mentioned authorities suggests the antiquity of the disease in question, and shows that the earlier cultivators of our profession, so far at least as its identity is concerned, were not without a full knowledge of its existence.

The application of the term Diabetes, however, to *all* chronic and excessive discharges of urine, I regard as most unfortunate; inasmuch as it has led, in many instances, no doubt, to malpractice—such discharges being treated as Diabetes by *name*, regardless of the *quality* of the urine voided, or of the *causes* producing it. The treatment, in the one case, if not productive of positive injury, can certainly produce no good results.

Passing, without special notice, the other, or all other varieties of this affection, I propose to confine myself to that form of the disease known and recognized as Diabetes Melletus; or, more properly, perhaps, Saccharine Diabetes, that form in which sugar, in greater or less quantities, is known to exist in the urine. Before proceeding further, did it not seem presumptuous in me, and as casting an imputation upon the intelligence of this Society, I might here pause, and invite your attention to the heterogeneous and compound characters of the urine, even in its most healthy condition.

An examination of healthy urine, deposited in a vessel, reveals to the eye no striking peculiarities; straw or amber colored fluid, specific gravity from 1010 to 1015, with rather a peculiar smell, is all that distinguishes it from any other fluid of a specific gravity from 1010 to 1015; to the senses it seems to be homogeneous. The most credulous could scarcely believe that some 14 or 15 substances or ingredients enter into its composition; several of which are chemically incompatible, yet so mixed and blended with each other, and in such harmonious proportions, as to constitute apparently one homogeneous whole. Sugar, however, is not one of the ingredients or elements of healthy urine. Though it is an element perhaps always present in the healthy animal economy, its appearance in the urine may be regarded as a messenger of evil omen, the harbinger of fearful portent; an enemy has insidiously crept into the citadel. The kidneys, those sleepless sentinels upon the watch-tower, that never know repose, have seized upon, and are endeavoring to eject him—eliminate him, if you please—as they do all other debris and effete matter, the retention of which would be destructive to health and life.

This disease, if such it can be called, from time immemorial has been regarded as among the *opprobria medicorum;* and, I believe, is one of the uncoveted legacies bequeathed to man only; no inferior animal, so far as I know or believe, ever being the subject of Saccharine Diabetes.

The justly celebrated Dr. Prout, to whose labors we are indebted for the existence of urinary pathology as a science, remarks: that animals are not subject to Saccharine Diabetes; and very pertinently asks, Can the exception be referred to that fertile cause of bodily disorder, *the influence of the mind?* M. Claude Bernard, the distinguished French physiologist and pathologist, mentions this fact, and almost laments it; as the physiologist, in consequence, has no means of vivisection.

Dr. Watson, the distinguished author of a Practice of Medicine, with which we are all familiar, says he had a coach-horse, that he supposed might have Diabetes: he was a great eater, and drank eagerly, but grew thinner and thinner. Dr. Prout, he says, was kind enough to examine his urine. It contained no sugar. In this connection, allow me to state that an eminent physician of this city informed me that he lost a fine milch cow of Diabetes; discharging large quantities of urine, from the effects of which she died. He thought it probable that it was saccharine—the weight of testimony, however, preponderates against this conclusion.

If, then, as has been conclusively shown, of all created animals, man only is the subject of this malady, as well as of other analogous affections—hysteria, for instance, which may be regarded as the analogue of Saccharine Diabetes, and of which the inferior animals are never the subjects—how are we to account for their origin, except in the more highly elaborated and exalted innervation? A brain and nerve development of such exquisite, delicate structure, that while they supply the physical machinery of our bodies with all the facilities and capabilities necessary to put us in a proper, pleasant, and useful relation with the world around us, they at the same time furnish a matrix, a home for the intellect, a temporary resting-place for the immortal mind—the Soul, if you please—which grows and expands, *parri passu*, with the developments of our bodies.

I pretend to no knowledge of that subtle and mysterious connection which exists between mind and matter. Such a disquisition would be regarded as metaphysical, and foreign to the subject before us. But because we do not *understand* it, it is no proof that such connection does not exist. Do we understand how vitality, *Life*, is connected with our physical bodies? We do know many of the causes that work its destruction; but of that close, intimate connection, the whys and wherefores of which we are ignorant; and, from the very nature of the subject, it is humiliating to believe we must ever remain so. That such connection does, however, exist, we have positive proof, we are not left to conjecture; and, it is equally certain that the dependence, one upon the other, is mutual—*sana mens in sano corpore* is a trite adge; as true, however, as it is trite. Inflict upon the body severe injury, and the mind presently suffers; and, *vice versa*, severe and protracted grief, disappointed love, heavy and irreparable losses of property, of friends, and many other mentally depressing causes, react injuriously upon the body, and probably first upon the stomach, and other digestive and assimilating organs; giving rise to indigestion, and, as a consequence, to malassimilation, and, as a sequence, defective nutrition, with a long train of what we call nervous symptoms.

The cravings of hunger are at once appeased by the receipt of greatly perturbing or distressing intelligence; protract this distress indefinitely, and disease will surely follow. If any predisposition exist, we may look for Saccharine Diabetes, or some other malady growing out of deranged or imperfect innervation. That Saccharine Diabetes then is a disease, or rather the symptom of a disease, the remote cause of which may be sought for in the nervous system, I think admits of no rational doubt: the assertion, however, of any hypothesis, no matter how plausible, without reason to sustain it, is an illogical and unsatisfactory mode of reasoning. I shall therefore be excused by the Society for trespassing upon its time, while I present the reasons, collaterally, remotely, and immediately, bearing, as I conceive, upon this subject. If it be admitted that it is of nervous origin, where, or in what part of the brain or cerebro-spinal system, shall we locate it? I answer, at the base of the brain (in the fourth ventricle), or perhaps in the medulla oblongata.

The nerves presiding over and controlling the functions of the kidneys, and their congeners, the procreative organs, have, by physiologists and phrenologists, by common consent, from time immemorial, been located, and not without reason, in that part of the brain. Who among us that ever heard that king among lecturers, Professor Caldwell, on Physiology and Phrenology (for he lectured on and taught both), that does not remember his phrenological illustration of the location of the amative and procreative organs, by reference, among many other examples, to the case of a man who, from the receipt of a blow upon the occiput, had priapism as the result, and whose venereal propensities became so inflamed, that he broke over all restraints of decency; and, unless forcibly restrained, would have committed a rape upon any person that wore the female attire, even his own daughters? This, too, from a blow over the cerebellum. Again, Dr. Errichsen says that he saw a case of Saccharine Diabetes supervene upon a slight contusion upon the back of the head of a man who fell from a hay-stack; it disappeared, however, upon a reparation of the injury. And just here, allow me to state what I witnessed some seventeen or eighteen years ago, in my own practice, as collaterally, at least, connected with this investigation. Early in the morning, I was sent for urgently to visit a young, stout, athletic negro man, the property of Mr. W. O. Hyde. In this fellow was fully developed all the points indicating his strong venereal propensities and lusts: short, thick, bull-neck; very prominent occiput; most of his brain behind his ears, etc. Upon reaching his house, which was very near the dwelling, I found him very much alarmed and greatly excited. He was the subject of priapism. The sword had been drawn, and he was unable to return it to its scabbard. My old preceptor's case at once suggested itself to my mind; and my first inquiry was, Have you struck the back of your head against any hard body, or in any manner hurt your head, or any part of it? He replied not. Do you feel any uneasiness about your head? He said that the back of his head hurt him. He then gave me, in the presence of his master, a full and frank statement of the facts of his case—and the cause of the priapism was apparent—he substantially stated as follows:—An addition had been made to the family, a day or two previously, of a young and likely girl, a house servant. They occupied the same room: the light of the apartment had scarcely grown dim, before he softly approached her couch, embraced her, wooed and caressed her, with all the soft and delicate expressions of love and affection that his amorous feelings could suggest. Not even the chivalrous and redoubtable knight of the broken helmet could have felt more deeply enchanted, when he mistook, in the enchanted inn, the embraces of a black, thick-lipped wench, for his own matchless and pearless *Dul Cenia del Toboso.* Our hero, however, wooed in vain: while she permitted such liberty calculated to inflame the ardor of his passions, she positively refused the only indulgence that could cool the incalescence of a passion which, before day, wrought itself up to a red heat. A little after the dawn of day, however, her hitherto inexorable determination yielded; and he had not more than fleshed his sword, before the sudden opening of the chamber door, and the voice of the mistress, so startled *her*, that suddenly springing up, she dismounted the hopeless knight, and left him musing upon the uncertainty of all sublunary affairs, and thoroughly impressed with the truth of the adage—that there is many a slip between the cup and the lip.

The facts of the case obtained, I immediately turned loose upon the bristling front of this great moral persuader, as the Irishman would call him, the most effective artillery at my command—the lancet, tartar emetic, *ad nauseam*, cold effusions to the back of the head and neck, were all called into active requisition, and were perseveringly played upon him for 12 or 14 hours. When unable to longer *stand* under the unequal contest, he capitulated, and lowered his colors, slowly and sullenly retiring within his breastworks; refusing, however, an unconditional surrender. For, even on the third day, he was, as the boys would say, in a big limber, still showing fight. Dropping the metaphor, this case did not fully recover until a large blister was drawn between the shoulders. The then owner, Mr. W. O. Hyde, resides within three miles of this city, and will vouch for the truth and accuracy of this statement.

What are the legitimate inferences to be drawn from this and similar cases? None other, than that disturbance of the nervous centres cannot long exist without derangement of the functions of the organs over which they preside; and the derangement will be in proportion to the intensity of the disturbing cause.

In the case just detailed, there was intense excitement, long continued, the most intense perhaps of all other excitements, producing an erethism of the nervous centres, that nothing but active treatment and time could quell: the erection was the effect of a mere symptom, violent reflex action; remedy the source—strike at the fountain, *fons et origo mali*, and the erection subsides; let both go unchecked, inflammation sets itself up in the virile organ, and perhaps mortification and gangrene will lop off an invaluable member: we see an analogous case in traumatic tetanus, in the unyielding rigidity of the muscles; modified, however, in this particular—the one is influenced by the excito-secretory, the other by the excito-motory system of nerves.

The reverse of this obtains; in Diabetes Melletus this disease is generally slow and insidious in approaches to the system; and, as I have ascertained from two of the sufferers, one of whom fell its victim, a total loss of virility, in the male, is among the first symptoms that give anxiety to the patient. Is not the remote cause of this disease in close proximity with the nerves of procreation, and by which they are, at least, sympathetically impressed?

This indifference to the whisperings of venereal indulgence cannot be charged to debility, for we know that in some other diseases (phthisis pulmonalis, for instance), where the debility and prostration are much greater, the venereal propensities, instead of being annulled, are, in fact, rather increased; and it is perhaps the last passion that yields to this voracious destroyer.

I have already alluded to the slow and almost imperceptible manner in which Saccharine Diabetes invades the system; and, with the views of its pathology already expressed, the Society are prepared to understand, first, that I regard it as the effect of combined physical and mental causes, operating upon the system indefinitely; and, secondly, that the disease rarely originates in any other way, except by hereditary transmission, and only then when the dormant susceptibilities are aroused by some exciting cause; mainly, I believe, by indulgence in improper diet. That the disease is hereditary, admits of no doubt. Dr. Prout informs us that he has seen and treated many cases of Saccharine Diabetes, that were clearly hereditary in their origin. Drs. Golding Byrd, Watson, and others, confirm this statement. Indeed, we need not go beyond the limits of our own little city for proof of the hereditary origin of this disease, as two distinguished members of this Society, now present, can testify. That the disease generally originates from wear and tear of mind and body, let an example or two be submitted. Some 12 years ago, there might be seen on College Street a stout, healthy, robust young man, with no hereditary taints about his system, engaged in the vocation of a livery-keeper. Family afflictions, of a severe character to a sensitive mind, overtook him. He purchased a farm in the country; abandoned the city and a life of ease for the sterner and more laborious pursuits of agriculture, in which he actively engaged himself, making a hand in the field. In a short time, the mental abrasions still going on, his health began to fail; medical advice was sought, and, at different times, running through a period of seven or eight years, he was treated by half a dozen or more of the most eminent physicians of this city. His health, however, still continued to go down: a visit to some fashionable watering-place was advised, with no benefit; rather an aggravation of his disease. Some three or four months before his death, circumstances threw him into my neighborhood. The character of his malady, by this time, was unmistakable. It was Saccharine Diabetes, beyond any doubt; but so insidious had been its march, so obscure its symptoms, that it eluded the notice of his physicians. The complications supervening, not long before his death, and which, most likely, would have attracted the attention of the uninitiated, were pulmonary tuberculosis, with convulsions; a very frequent termination of the malady, if we may trust the authorities; the organ last attacked indicating the original seat of the disease. This case is an instructive one. It was at no time characterized by the excessive discharges of which we read, and, indeed, generally witness in the affection; had it been, there would have been no difficulty in correctly diagnosing it, in the beginning. The amount of urine passed was not greatly over the normal standard; but, I have no doubt from his statements, that it had been Saccharine from the beginning.

One more case, gentlemen, and I will dismiss this branch of the subject. A gentleman well known in this community, of rather delicate physical structure, of sanguine temperament, of very active and industrious habits, and who accumulated an estate by his energy and indefatigable attention to the business and duties of an office that required unceasing mental as well as physical toil. Under such protracted exertion, his digestion became impaired, and Saccharine Diabetes supervened, as a consequence; in a few months, he was numbered among the victims of that fell destroyer.

Here we see all the cases in full operation, aided, perhaps, by inattention to proper dietetic regulation, to wake up the susceptibility, the predisposition that might otherwise have remained in abeyance in his system.

And here, gentlemen, allow me to ask, Did ever a case of Saccharine Diabetes fall under your observation that could not be traced to antecedent causes, such as I have described; excepting, always, such as are of hereditary transmission? And, second, have you ever discovered a case in the Negro? If not, why is he exempt from it? Let Ariel answer.

Having now, though in a very imperfect and unsatisfactory manner, I confess, settled the pathology of the disease, the remote pathology, it may be expected that I should say something of its proximate cause. How and where is the sugar formed? What particular organ or organs elaborate it? And, first, as to the proximate cause, let the illustrious Prout—though dead, he still speaketh, and will speak as long as urinary pathology shall have a votary—he says: the proximate cause of Diabetes is exceedingly obscure; that if you will tell him how fat is formed in the animal economy, or how bile is formed in the liver, he will tell you how sugar is formed in the body. The same distinguished man, in the first edition of his book on Urinary Pathology, says: it is formed in the kidneys. He subsequently abandoned that ground, and located its formation in the *primæ viæ*. The experiments and observations, however, of still later pathological investigators, locate it in the liver. Among the latter may be mentioned the names of Monsieur Bernard, of Paris, an announcement which he made about the same time that Marshall Hall made and announced the discovery of a second system of nerves, which he named the excito-secretory;* having previously announced the discovery of the excito-motory. Dr. Hall's second announcement of the excito-secretory system

*And before this announcement, Professor Campbell, of Georgia, had already announced the same thing. Dr. Marshall Hall, in an autograph letter to Dr. Campbell, concedes to the latter priority of claim to the discovery, and gracefully adds:—"The field is indisputably your own."—[Ed.]

was based upon what he styled "the brilliant experiments of Monsieur Bernard," and particularly his experiments upon the pneumo-gastric nerve, in relation to the secretions of the liver. It will be remembered by this Society, that our own countryman, Henry F. Campbell, of Georgia, claimed priority in this discovery, and opened a correspondence with Marshall Hall, in reference to it. How the matter was adjusted between them, or what the award of the literary world was, I am unable to say. But, to return from this digression, Dr. Bernard's experiments were reported by Dr. Pavy, of London, a gentleman whose fame as an able pathologist is second only to Bernard's. Dr. Davis verified and confirmed the experiments of Bernard, and pushed them still further; thereby furnishing to our profession a noble example of justice and magnanimity, of giving to Cæsar the things that are Cæsar's. The contempulle really too frequently existing between master minds of the same locality, and especially of different nationalities, too often distorts, and seeks to detract from the merits of each other. Not so, however, with Hall, Pavy, and Bernard. In the same great field, as yet but little explored, the trio march forward hand in hand; and, while enriching science with their discoveries, they are making for themselves imperishable fame.

But to return: a few of the conclusions at which these distinguished men have arrived, based upon observation and experiments, are, that the liver, during life, produces no sugar, but only the substance, named by Bernard, glucogen, but which Dr. Pavy prefers to call hepatine; now, however, he calls it amaloid, and it is only under peculiar circumstances in the living subject that it becomes sugar: the sugar found in the liver and blood, after death, is a *post-mortem* change of hepatine. Cane sugar administered appears as grape sugar in the urine. Hepatine is not naturally formed for transformation into sugar. It is probable, they say, that an altered state of the blood can produce the change by which hepatine passes into sugar: phosphoric acid, injected into the jugular vein, produces sugar, by destroying the alkalinity of the blood. The saliva, blood, and tissue of liver transform hepatine into sugar most readily. Contact with saliva, at 100 Fah., almost instantaneously. Dr. Pavy believes that hepatine goes to the formation of fat; that it does not enter the blood, as hepatine or glucogen, may be inferred from the fact that when that substance is mixed with the blood, it immediately becomes sugar. Dr. Pavy believes, with Bernard, that sugar taken into the stomach is not absorbed by the lacteals, but passes into the portal circulation; in the liver it will appear to become hepatine, and to undergo some other change before it enters into the system. The views formerly entertained, and in which Dr. Pavy concurred, as to an extensive destruction of the saccharine principle, especially taking place during the passage of the blood through the lungs, are now shown to be incorrect. I regret, gentlemen, that I have not the time to pursue this subject further, but would refer those desiring further information to a little book recently published by Dr. Jno. Camplin, of London. With the single expression of my own poor opinion, that the sugar is formed in the *primæ viæ*, from causes already detailed, passes thence into the blood, and eliminated by the kidneys.

I now pass to a detail of the cases promised, with their treatment, as briefly as circumstances will allow me, having wearied the Society, I fear, with a paper much longer than was intended.

Case I. Mr. C. G., a citizen of this city, aged about 56, very black hair and eyes, dark and florid complexion; cabinet workman by profession, sought medical advice from me, a little more than 12 months ago. I had known Mr. Gower for many years; lived near me when I resided in the country: from being a stout and very active man, he was so reduced and enfeebled that he excited the sympathies of all who knew him. He tottered as he walked; he did not attempt to walk unless supported by his cane. He was free of cough, or I should have supposed him far gone in consumption.

He told me that for about three years he had been troubled with frequent and heavy discharges of urine. The Doctors called his disease "Diabetes." He had consulted with several medical gentlemen, and had been under treatment for more than two years, but had steadily gone down until he could hardly put one foot before the other. "How much water do you pass in twenty-four hours?" "From 16 to 18 pints." "Have you *measured* it?" "I have." "Have you been passing that quantity all the time?" "Well, I don't know that I have every day and night, but I pass a great deal all the time." "What is the color of your water?" "About the common color—rather deeper at some times than others; and, as I pass it in a vessel, seems thick, and foams like soap-suds." "Have you ever tasted it, or had reason to believe that it contained sugar?" "I have; the Doctors told me that there was sugar in it. I know it has, for the flies swarm around it, as they do about a leaky molasses barrel; and, when passing it, a drop that chances to fall upon my pants, upon drying, becomes of a whitish cast; or, if I make water upon the grass about the yard, upon drying, it leaves a whitish-looking deposit." No chemical tests or analyses were necessary to assist the diagnosis in this case. It was beyond doubt a case of Saccharine Diabetes, and, as I thought, beyond remedy. He was troubled, also, with tormenting thirst and a voracious appetite, and at night especially, with horrible pains of the loins, hips, and legs. I commenced, after giving him strict injunctions as to diet; directing him to refrain from, as far as practicable, all food of an amylaceous character. I put him on creasote, four-sixths of a drop diluted in water, to be taken during the day and night, with opium, *pro re nata*, from two to four grs. per day, with various plasters, unguents, liniments, etc., to the different seats of pain. Passed the electric current several times through his emaciated and dwindled lower extremities. Under this treatment the disease was mitigated and held in abeyance. The quantity of urine diminished; and in some respects his case was more hopeful; but he gained neither flesh nor strength. Thus stood the case until the first of January last, about which time I saw in *The New York Medical Record*, a notice of a new work on Diabetes Melletus, by Dr. John Camplin, of London, himself a diabetic. The book bears the title: "On the Jurantia et Sedentia in Diabetes." I lost no time in procuring a copy, which I did in a few days, through the kindness of Messrs. Paul, Tavel & Hanner, booksellers of this city. Gave it a cursory examination, and, as speedily as possible, adopted the treatment to the letter indicated by Dr. Camplin. The change in my patient was not only unexpected, but I almost doubted the possibility that in so short a time dietetic regulations only could have wrought such a change. I regarded it either as a miracle, or one of those freaks in disease now and then met with, where apparently rapidly-approaching death lets go its grip, and lets the prisoner free, notwithstanding the prognosis of the Doctor. In three days, Mr. G. will tell you, his urine diminished at least one-half; his pains, so agonizing before as to require opium, left him, as by enchantment; his mouth and tongue, before dry, became moist; salivary glands discharged their functions well; relished his chew of tobacco, and spit as well as anybody; his spirits, before gloomy and depressed, became buoyant and cheerful; and, in eight weeks from the time he commenced the treatment, he had gained 8 pounds, or 1 pound a week. By this time could chop his own wood, get his own water, walk the distance of nearly a half mile to market, with basket on arm, and back, without fatigue.

This is a truthful and correct account of the progress of this case under Dr. C.'s treatment. What treatment is it that can almost raise the dead? What raised the Doctor himself (who was almost at death's door) from the same disease; and, as he avers, not only mitigated, but cured a number of cases, almost as far gone as his own? The character of the man, as set forth in his writing; his associations with such men as Dr. Prout, whom he consulted, and who suggested the bran-cake as a proper diet for him, taken altogether, leave us no doubt of the truth of his statements; more especially, when, to a great extent, we have verified them to the letter; and found that whenever the patient violated the programme of diet, the injurious effects would be manifested in less than ten hours.

The treatment is mainly dietetic, and consists in a total abnegation of all and every species of amylaceous food. Touch not, taste not, handle not, *starch.* It is an unclean thing in this disease. As soon as swallowed it is converted into sugar; and in proportion to the quantity taken will be the amount of sugar manufactured; which excites the kidneys; and the flood-gates are open; and wasting and exhausting discharges drain the body of flesh and strength, besides deranging almost every function. Dr. Prout suggested to Dr. C. the bran-bread or cake: Complin tried it, with no good results, because it contained 52 per cent. of starch, as an analysis proved. Complin then determined to have the starch washed out, which he did, by passing it through two or three waters. That done, it was dried in a quick-oven and pulverized; then milk, eggs, soda (or not), ginger or nutmeg, and the whole kneaded with butter and baked in small pans, quickly, make up the Camplin bran-cake; which, as it contains no starch, may be eaten with impunity by the patient, with butter, mutton, pork, cheese, milk, and soft eggs. Vegetables are permitted, of the non-amylaceous family. My patient ate freely of cabbage and sour-krout during the winter, and no bad results were noticed after it. As to medical treatment, I have but little to say. No therapeutic agent, I believe, it is conceded, exerts much influence over the formation of the sugar; and any medicine or food that cannot or will not do that, will be either nugatory or mischievous. Medicines may be, and no doubt are necessary, *pro re nata*, during the existence of the disease. The liver, the skin, the bowels and lungs, should in this, as in all diseases, share our attention.

Case II. This case is a sprightly, intelligent young lady of this city, who, while in the city of New York, on a visit, last winter, had an acute attack of hereditary Saccharine Diabetes. I say hereditary. Her father died of the same malady, about two years ago. Her friends had her brought home here. Dr. Atchison, the family physician, aided by Dr. Joseph Jones, was in charge of her case, when I also was invited to see her. Finding that she was upon the Camplin treatment, already detailed in case 1st, I retired, leaving the case in the care of the above-named gentlemen.

Dr. Atchison, at my request, furnished me, a few days since, the following statement in reference to the treatment, results, etc., of the case, *viz.*:—

DEAR DOCTOR:—The following is a brief outline of the case of Diabetes Melletus, now under my care:—A young lady, aged 18, light hair, fair skin, small stature, having previously enjoyed good health, was attacked about the 1st of January, while on a visit to the city of New York, with what was at first supposed to be by her physicians remittent fever, characterized by great thirst, dry tongue, febrile exacerbations in the evening, loss of appetite, etc. After two or three weeks of unavailing treatment, attention was called to the urinary organs, and an analysis made of the urine, which revealed the following results: —The amount of urine passed in 24 hours, 5½ pints; sugar per ounce, 5 grains; specific gravity, 1038. She was immediately ordered on ferruginous tonics, nitric acid, and the Camplin diet. Her friends being informed of her condition, she was ordered home: treatment was not commenced until the 16th of February, when, assisted by Professor Joseph Jones, another analysis was made, showing: 7 grs. sugar to the ℥j., with a specific gravity of 1040; quantity passed, 7½ pints per day.

No appearance of catamenia for three months. Bowels constipated and distended; countenance pale; digestion feeble. Ordered pills, Vallet's mass., co. extract colocynth, ex. nux vomica, to be given three times a day, with cod-liver oil, and the Camplin diet to be rigidly adhered to, with saline baths and free exercise in the open air. No material change for about two weeks. Quantity of urine per day varying from 5 to 7 pints. At this time a large amount of scybalous matter was discharged from the bowels, with great relief. Appetite and general appearance improved. Specific gravity of urine fallen to 1020; quantity not materially lessened. Pills and cod-liver oil withdrawn; ordered phosphates of soda, lime, and iron, to be given in milk, after each meal. Diet continued, and visit to the country. In about three weeks she returned much improved, voiding only about 3 pints per day. No analysis made. Has gained strength, and general appearance good. Treatment continued, and again ordered to the country. From that time the convalescence was uninterrupted. Menstrual flux occurred on the 3d of May, normal in quantity, and without disturbance. Quantity of urine now passed per day, about 2 pints, healthy in appearance. Bread is now allowed in moderate quantities. She has resumed her natural gaiety and *embonpoint*, and seems perfectly well; though, as a prudential measure, her diet is still much restricted.

In conclusion, gentlemen, I will add, that when either of these patients departed from the programme of diet, the bad effects were clearly observed in 8 or 10 hours; the amount of urine being greatly increased; and, I have no doubt, the increase was due entirely to the conversion of the starch into sugar, which stimulated the kidneys to increased secretion.—*Nashville Journal of Medicine and Surgery.*

